# Circadian regulation of abiotic stress tolerance in plants

**DOI:** 10.3389/fpls.2015.00648

**Published:** 2015-08-27

**Authors:** Jack Grundy, Claire Stoker, Isabelle A. Carré

**Affiliations:** School of Life Sciences, University of Warwick, Coventry, UK

**Keywords:** circadian clock, rhythmic gene expression, abiotic stress, plant hormones

## Abstract

Extremes of temperatures, drought and salinity cause widespread crop losses throughout the world and impose severe limitations on the amount of land that can be used for agricultural purposes. Hence, there is an urgent need to develop crops that perform better under such abiotic stress conditions. Here, we discuss intriguing, recent evidence that circadian clock contributes to plants’ ability to tolerate different types of environmental stress, and to acclimate to them. The clock controls expression of a large fraction of abiotic stress-responsive genes, as well as biosynthesis and signaling downstream of stress response hormones. Conversely, abiotic stress results in altered expression and differential splicing of the clock genes, leading to altered oscillations of downstream stress-response pathways. We propose a range of mechanisms by which this intimate coupling between the circadian clock and environmental stress-response pathways may contribute to plant growth and survival under abiotic stress.

## Introduction

The world-wide population is predicted to increase from 7.2 billion to between 9.6 and 12.3 billion by 2100, increasing the demand on global food productivity (Gerland et al., 2014). However, nutrient deficiency, mineral toxicity and salinity severely limit the amount of land that is available for food production. In addition, world-wide crop losses due to extreme temperatures, drought, and flooding are estimated to rise about 50% by 2050 (Boyer, 1982). Water deficit was estimated to have caused €100 billion worth of damage in Europe between 1977 and 2007 (European Commission, 2007). The severe drought of 2012 affected 80% of the crop-growing territory in the USA (Hoerling et al., 2013), and such droughts are predicted to become more frequent as a result of global warming (Hansen et al., 2010; Lawrimore et al., 2011; Lobell and Gourdji, 2012; Cook et al., 2015).

There is an urgent need for development of crop varieties with improved tolerance to abiotic stress, but the development of such improved varieties will require a thorough understanding of the mechanisms by which plants are affected by these conditions, and how they can tolerate them. Abiotic stress can affect cellular homeostasis via a variety of mechanisms. Freezing temperatures promote intra- and extra-cellular ice crystal formation, leading to cellular damage, dehydration and disruption of the plasma membrane (Steponkus and Webb, 1992; Pearce, 2001; Yamazaki et al., 2008, 2009). High temperatures damage the plasma membrane by increasing its fluidity, leading to ion leakage. Heat stress can also result in inhibition of enzymatic activity and photosynthesis (Salvucci and Crafts-Brandner, 2004; Sharkey, 2005; Wahid et al., 2007; Allakhverdiev et al., 2008). High temperatures, salinity and drought stress are often accompanied by production of reactive oxygen species (ROS) which can lead to denaturation of functional and structural proteins (Gill and Tuteja, 2010; Suzuki et al., 2012). Gradual exposure to these different stresses causes plants to become more tolerant, however, and acclimated plants can survive harsher conditions than non-acclimated plants. The acclimation process includes the expression of new transcripts and proteins, and the accumulation of metabolites that result in a new homeostatic state that is more compatible with the change in environmental conditions. This allows stabilization of proteins and membranes, osmotic adjustment and re-establishment of the redox balance (Krasensky and Jonak, 2012; Mittler and Blumwald, 2015).

A number of transgenic lines with improved tolerance to environmental stress have been generated in a variety of crop species, and a few of these have been approved for commercialization (Lawlor, 2012; Cabello et al., 2014; Waltz, 2014). These transgenic lines exploit a variety of transgenes that act to maintain osmotic balance under abiotic stress conditions. For example, Monsanto’s DroughtGard maize (MON87460) was transformed with the cold-shock protein CspB from *Bacillus subtilis* to improve water efficiency under drought stress (Nemali et al., 2014). The *betA* gene from the bacterium *Rhizobium meliloti* was introduced into Indonesian sugar cane in order to increase production of the osmoprotectant glycine betaine, resulting in enhanced resistance to drought (Marshall, 2014). An osmoregulator protein (osmotin) from tobacco was also used to improve the salinity tolerance of chili pepper, cotton, tomato, and strawberry plants (Husaini and Abdin, 2008; Parkhi et al., 2009; Goel et al., 2010; Subramanyam et al., 2010; Patade et al., 2013).

While these approaches have led to significant improvements, the genetic manipulation of master regulators of stress responses would potentially be more powerful because of the broader spectrum of resistance mechanisms that can be triggered by such modifications. However, as natural stress responses often lead to growth arrests, such modifications often cause detrimental pleiotropic effects, leading to reduced yields under normal conditions (Hussain et al., 2011). These problems can be alleviated by the use of stress inducible promoters, such that the genetic modification is only expressed at times when it is beneficial. Alternatively, the judicial choice of transcription factors that control specific pathways with minimum unwanted side effects can minimize such issues (Hussain et al., 2011). Nevertheless, an ideal approach to improving crop performance under abiotic stress conditions would be to exploit a genetic pathway that (i) contributes to tolerance to a broad range of environmental stresses and (ii) can naturally adapt in to enhance stress tolerance mechanisms when plants are exposed to deleterious conditions. Here, we will discuss recent evidence that the plant circadian clock may represent such a mechanism.

Circadian clocks are 24-h biological oscillators, which generally enable organisms to anticipate predictable, daily changes that are linked to the rotation of the earth. Such clocks have been found in a wide range of organisms, including cyanobacteria, fungi, insects, and mammals (Young and Kay, 2001). Having a clock is not essential for plant viability, and arrhythmic mutants are still capable of developing (Green et al., 2002). However, there is convincing evidence that having a clock that is well tuned to diurnal cycles of light and darkness makes an important contribution to plant fitness. For example, plants whose clocks were a good match to their rhythmic environment performed best in competition experiments (Dodd et al., 2005). This may be explained, at least in part, by the role of the clock to ensure the optimum use of starch at night while avoiding starvation before dawn (Graf et al., 2010; Scialdone et al., 2013; Mugford et al., 2014; Webb and Satake, 2015). However, recent data suggest that the circadian clock may also contribute to plant fitness by enhancing their ability to tolerate abiotic stress.

## Abnormal Function of the Circadian Clock Results in Altered Abiotic Stress Tolerance

The molecular mechanism of the plant circadian clock was investigated using the genetic model system *Arabidopsis thaliana*, and was found to be composed of a transcriptional regulatory network. The mechanism by which this network oscillates has been reviewed in detail elsewhere (see, for example Carré and Veflingstad, 2013; Hsu and Harmer, 2014; Seo and Mas, 2014), and we will only describe it minimally here, to introduce the relevant genetic components. Briefly, the day starts with expression of two closely related transcription factors, LATE ELONGATED HYPOCOTYL (LHY) and CIRCADIAN CLOCK ASSOCIATED 1 (CCA1), which peak in the early morning (Schaffer et al., 1998; Wang and Tobin, 1998). Transcription of the *LHY* and *CCA1* genes is down-regulated in the afternoon and through the night by a set of sequentially expressed PSEUDO-RESPONSE REGULATOR proteins known as PRR 9, 7, 5, and 1, where PRR1 is also known as TIMING of CAB or TOC1 (Matsushika et al., 2000; Strayer et al., 2000; Alabadi et al., 2001; Nakamichi et al., 2010). The inhibition of *LHY*/*CCA1* transcription by the PRR proteins is released late at night through the action of an evening complex (EC) composed of three proteins, EARLY FLOWERING (ELF) 3, ELF4, and LUX ARRHYTHMO (LUX). The EC shuts down the expression of the *PRR* genes, which allows expression of *LHY* and *CCA1* to rise again in the following morning (Hicks et al., 2001; Doyle et al., 2002; Hazen et al., 2005; Onai and Ishiura, 2005; Nusinow et al., 2011; Pokhilko et al., 2012). LHY and CCA1 subsequently bring down expression of *TOC1* and the cycle restarts (Alabadi et al., 2001). The F-box proteins ZEITLUPE (ZTL) and LOV KELCH PROTEIN 2 (LKP2), function as a complex with GIGANTEA (GI) to control the light-dependent proteolysis of the TOC1 protein, and this acts to increase to amplitude of oscillations of the gene network under diurnal light–dark cycles (Mas et al., 2003; Kim et al., 2007). The function of the central oscillator is synchronized to environmental light dark cycles via input pathways mediated by phytochrome and cryptochrome photoreceptors (Somers et al., 1998). The circadian clock in turn controls downstream physiological processes via a number of mechanisms, termed output pathways.

Mutations in components of the circadian clock not only impacts on the function of the central oscillator but also alter plants’ ability to both tolerate environmental stress conditions, and acclimate to them. For example, plants with mutations in *CCA1*, *LHY*, *ELF3*, *ELF4*, *LUX*, *PRR5*, *PRR9*, and *PRR7* are hypersensitive to ROS-generating agents, whereas *CCA1*-overexpressing plants (*CCA1-ox*) are more tolerant than wild-type plants (Lai et al., 2012). Overexpression of *LKP2* enhances drought tolerance (Miyazaki et al., 2015). The *prr5,7,9* triple mutant is more tolerant to high salinity and drought stresses (Nakamichi et al., 2009). *GI-ox* plants show enhanced salt sensitivity, whereas *gi* mutants show enhanced salt tolerance and enhanced survival under drought stress and oxidative stress (Kim et al., 2013; Fornara et al., 2015). Reduced expression of *TOC1* in *toc1-RNAi* plants results in improved drought tolerance, whereas overexpression of *TOC1* causes increased water loss and reduced survival under drought conditions (Legnaioli et al., 2009). The *prr5,7,9* and *toc1* mutants show significantly increased freezing survival (Nakamichi et al., 2009; Keily et al., 2013), but *gi*, *lux*, *lhy*, *cca1*, and *lhy cca1* mutants have reduced constitutive and acclimated freezing tolerance (Cao et al., 2005; Espinoza et al., 2010; Dong et al., 2011; Chow et al., 2014).

These findings demonstrate that the circadian clock contributes to plants’ ability to tolerate environmental stress. While the effects observed may seem relatively small (only a 1–2°C difference in freezing tolerance for *lhy cca1* double mutants, for example), this may prove valuable to allow recovery after a frost or after extreme drought for example (Lawlor, 2012). Interestingly, there is no correlation between the effect of mutations on the function of the clock and their effects on abiotic stress tolerance. For example, mutations which cause a short-period phenotype (*cca1*, *lhy*, *prr5*), a long-period phenotype (*prr9*) or complete arrhythmia (*elf3*, *elf4*, *lux*) cause similar hypersensitivity to ROS-generating agents (Green and Tobin, 1999; Mizoguchi et al., 2002; Ito et al., 2003; Yamamoto et al., 2003; Lai et al., 2012). Furthermore, plants that show similar arrhythmic phenotypes can have opposite phenotypes with regard to ROS sensitivity (*elf3*, *elf4*, and *lux* mutants vs *CCA1-ox*), drought tolerance (*LKP2-ox* vs *prr5,7,9* mutants) or freezing tolerance (*lux* vs *prr5,7,9* mutants; Hicks et al., 1996; Wang and Tobin, 1998; Schultz et al., 2001; Doyle et al., 2002; Hazen et al., 2005; Nakamichi et al., 2005, 2009; Chow et al., 2014; Miyazaki et al., 2015). This suggests that the effect of these mutations on circadian period or on the ability of the clock to oscillate is not the primary cause of the altered stress tolerance phenotype. A more likely hypothesis is that the altered expression level of the clock genes in the mutants has a direct impact to either up- or down-regulate abiotic stress responses, as discussed in the following sections.

## Circadian Regulation of Stress-Responsive Genes

The effects of the clock on plant physiology and development are largely mediated through the regulation of gene expression. Approximately 80% of transcripts cycle in *Arabidopsis* plants under diurnal light–dark or temperature cycles, and 30–40% of these oscillations persist upon transfer to constant conditions (Harmer et al., 2000; Edwards et al., 2006; Covington et al., 2008; Michael et al., 2008). Similar observations have been made in a number of crop plants and tree species, including rice, poplar, maize, tomato, and soybean (Facella et al., 2008; Khan et al., 2010; Filichkin et al., 2011; Marcolino-Gomes et al., 2014). Remarkably, the phasing of expression for the majority of genes was found to be conserved across monocots and dicots (Filichkin et al., 2011). One particularly interesting outcome from transcriptomic analyses was that many genes that are responsive to abiotic stress were found to be under the control of the circadian clock. Thus, in *Arabidopsis*, approximately 50% of genes that are responsive to heat, osmoticum, salinity or water deprivation were found to be expressed rhythmically in constant light, and 40% of cold-regulated genes (Covington et al., 2008). Rhythmic expression of abiotic stress-responsive genes was also reported for soybean and barley (Habte et al., 2014; Marcolino-Gomes et al., 2014).

**FIGURE 1:**
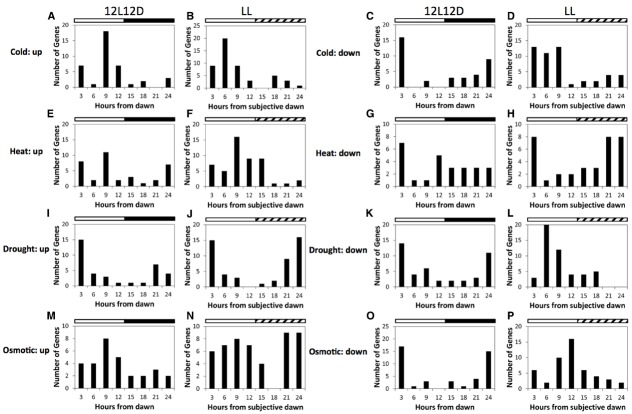
**Circadian regulation of (A-D) cold, (E-H) heat, (I-L) drought and (M-P) osmoticum-responsive genes in ***Arabidopsis***.** Histograms show the timing of peak mRNA levels in 12L12D or following transfer to constant light, for the 50 most significantly stress-induced (up) or repressed (down) genes that also exhibit circadian regulation. Lists of stress-responsive genes were based on the following datasets: 24 h at 0°C (Lee et al., 2005), 3 h of at 38°C (Barah et al., 2013), water limitation for 4 days (Wilkins et al., 2010), and growth on 300 mM mannitol (Kilian et al., 2007). Phase data for *Arabidopsis* genes were obtained from experiments LDHH-Stitt and LL12 in the Diurnal database (Blasing et al., 2005; Mockler et al., 2007; Covington et al., 2008). Solid white and black bars indicate intervals of light and darkness, respectively, whereas hatched bars indicate subjective nights.

This raises the question of how the daily timing of expression of these genes (i.e., their phase) relates to rhythmic changes in environmental conditions. This was examined in Figure 1, based on phase data for *Arabidopsis* genes retrieved from the Diurnal database (Blasing et al., 2005; Mockler et al., 2007; Covington et al., 2008) and on lists of cold, heat, drought and mannitol-responsive genes derived from existing datasets (Lee et al., 2005; Kilian et al., 2007; Wilkins et al., 2010; Barah et al., 2013). This showed that although individual genes within abiotic stress-response pathways were expressed across a broad range of phases, peaks of expression were clustered around specific times of the day. This was true for responses to environmental conditions that vary in a diurnal manner, such as cold or heat, but also for responses to conditions that progress over a number of days, such as drought, or conditions that are constant, such as high osmoticum. This suggests that circadian regulation of abiotic stress responses may confer an advantage to the plant even under conditions that do not change in a diurnal fashion.

**FIGURE 2:**
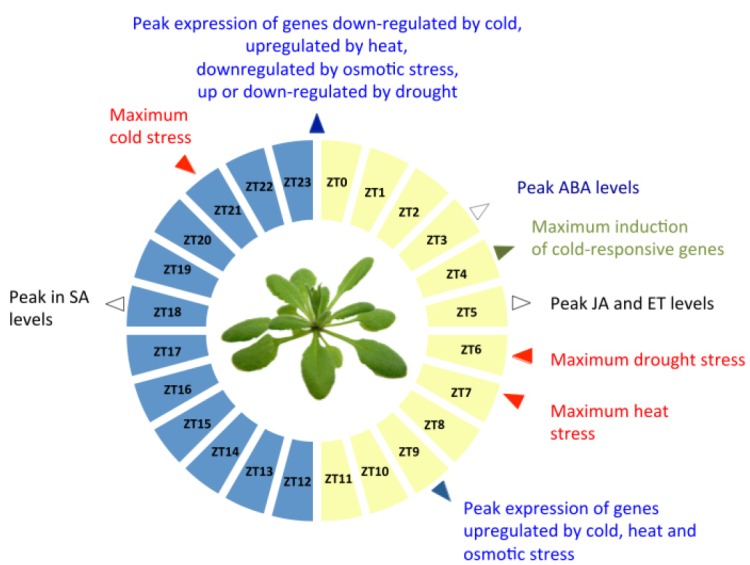
**Timing of abiotic stress responses across the day-night cycle.** The times of the day at which different types of abiotic stress are most prominent in the natural environment are indicated in red. For each type of environmental stress, the time of peak basal expression for the majority of stress-responsive genes is indicated in blue, the time of maximum accumulation of stress response hormones is shown in black, and the time of maximum responsiveness to different environmental signals is shown in green.

The majority of cold-inducible genes peaked in the afternoon, a few hours ahead of cooler temperatures at night (Figure 2). This included the cold-responsive transcription factors *CRE/DRE-BINDING FACTOR*s 1,2, and 3, which function as master regulators of the cold acclimation response (*CBF1*,*2*,*3*, also known as *Dehydration-Responsive Element Binding* or *DREB1–B*, *C*, and *A*, respectively), and the circadian and cold-regulated genes *CCR1*, *CCR2*, and *RD29A* (Carpenter et al., 1994; Harmer et al., 2000; Dodd et al., 2006). On the other hand, genes that are down-regulated by cold peaked around dawn (Figure 2). Thus, the timing of expression of cold-responsive genes was consistent with a role in anticipation of predictable, diurnal changes in temperature. In cotton and soybean plants, maximum low-temperature resistance was observed at the beginning of the night, corresponding to the time when most cold-responsive genes are expressed in unstressed conditions (Couderchet and Koukkari, 1987; Rikin et al., 1993). This suggests that rhythmic transcription of cold-responsive genes may form the basis of rhythmic changes in chilling tolerance.

In *Arabidopsis*, the majority of heat-inducible genes were expressed during the day, and heat-repressed genes were broadly expressed at night (Figure 2). Similarly, in spinach, five genes encoding heat shock proteins (HSP70s) were found to exhibit maximum expression in the middle of the day. This was associated with a diurnal variation in thermo-tolerance. Plants exhibited minimum heat-induced electrolyte leakage following treatments in the afternoon and early evening, which was consistent with the hypothesis, that the rhythmic expression of HSP70s confers heat tolerance at the end of the day (Li and Guy, 2001). The production of ROS-scavenging enzymes also peaks mid-day in *Arabidopsis* and may contribute to heat tolerance by minimizing of heat-induced oxidative stress (Lai et al., 2012).

The majority of drought-inducible genes peaked around dawn (Figure 2). Plants experience a water deficit in the daytime, due to the prolonged opening of stomata for photosynthetic gas exchange (Jarvis, 1976), and the rhythmic expression of drought-inducible genes may allow plants to minimize water loss at this crucial time. However, many drought-repressible genes were also found to peak during the day, when they would have been expected to peak at night. The physiological significance of this is unclear. In summary, while the timing of expression of stress-responsive genes is mostly consistent with the timing of daily environmental changes, this is not always the case. One possible explanation is that, genes which play a role in tolerance to a given type of abiotic stress may also contribute to other pathways, and that there may be a greater selective pressure for optimal timing of their expression relative to these other processes.

## Circadian Gating of Abiotic Stress Responses

In addition to driving the rhythmic, basal expression of stress-responsive genes, the clock controls the amplitude of their responses to environmental signals, in a process called gating. For example, cold treatments at different times of the day result in differential induction of *CBF1,2,3* transcription, with maximal induction observed in the morning (Fowler et al., 2005; Thomashow, 2010). Consistent with the role of CBF transcription factors as master regulators of the chilling response, about 75% of cold-responsive genes exhibit similar gating of cold responses with maximum responsiveness in the morning. A similar timing of maximal cold-responses was observed in peach trees. Out of five *CBF* homologs, four showed maximal cold-induction in the morning, and this was associated with induction of downstream targets including *DEHYDRIN* (*DHN*) *1* and *3* (Artlip et al., 2013).

The conserved gating of low temperature responses across distant plant species strongly suggests that the circadian gating of chilling responses confers a selective advantage. Little is known, however, about how the gating of temperature responses contributes to plant fitness. One hypothesis is that, while the rhythmic expression of cold-responsive genes allows anticipation of predictable cold temperatures at night, gated responses may be important to allow rapid changes in gene expression in response to unexpectedly cold mornings.

Responses to water deficit are also gated by the circadian clock, and maximal drought-induced changes in gene expression are observed at dusk (Wilkins et al., 2010; Kiełbowicz-Matuk et al., 2014). Interestingly, different sets of genes are induced by identical cold or drought treatments given at different time of the day (Bieniawska et al., 2008; Wilkins et al., 2010). Similarly, significant differences in heat stress responsive pathways were observed in grapevine, depending on whether the treatment was applied during the day or during the night (Rienth et al., 2014), and the physiological implications of these time of the day-differences remain to be investigated.

## Regulation of Abiotic Stress Responses by Core Oscillator Components

A number of components of the circadian clock encode transcription factors, which have a dual role, both to ensure the function of the central oscillator and to control the rhythmic expression of downstream genes.

Thus, a number of abiotic stress-responsive genes are under the control of core components of the circadian oscillator. For example, the LHY and CCA1 transcription factors play a key role to regulate low temperature responses by controlling expression of the *CBF* genes. The promoters of *CBF1*, *2*, and *3* contain LHY and CCA1 binding sites, also known as Evening Elements or EEs (AAATATCT). A CCA1:GFP fusion protein was found to bind the *CBF1-3* locus in chromatin immunoprecipitation experiments (Harmer et al., 2000; Dong et al., 2011), and loss of LHY and CCA1 function in the *lhy cca1* double mutant resulted in arrhythmic basal expression of *CBF1*, *2*, and *3.* This provided strong evidence that LHY and CCA1 play an essential role in the rhythmic expression of the *CBF* genes and of the cold responses that they regulate. The TOC1 and LUX proteins were also found to bind the promoter of *CBF3*, and mathematical modeling showed that the regulation of *CBF3* transcription by TOC1 and LUX was important for the correct gating of *CBF3* responses (Keily et al., 2013). Accurate gating patterns could only be reproduced in simulations when (i) expression of *CBF3* was down-regulated by TOC1 and the EC; (ii) it was up-regulated by LHY/CCA1, and (iii) cold was assumed to increase the level of LHY/CCA1 protein. In addition to controlling master regulators of the low temperature response, LHY and CCA1 control expression of cold-responsive genes in a direct manner. Thus, cold-responsiveness of the cold-inducible genes *CONSTANS-LIKE 1* (*COL1*) and *COLD REGULATED GENE 27 (COR27)* was shown to result from a synergy between EEs and ABA-responsive elements (ABREs, CACGTG) in their promoters (Mikkelsen and Thomashow, 2009).

LKP2 was recently suggested to play a role to control responses to drought. LKP2-overexpressing plants exhibited a smaller stomatal opening and showed elevated expression of several drought-responsive genes (Miyazaki et al., 2015). LKP2’s protein partner, GI was also found to regulate the expression of genes involved in drought and cold stress responses, including a number of *EARLY RESPONSIVE TO DEHYDRATION* genes (*ERD10*, *ERD7*, and *AT4G04340*) and cold-regulated genes (*COR78*, *KIN2*, *COR15a*, *COR15b*, *COR314,* and *COR413*; Fornara et al., 2015). This effect of GI was dependent on CYCLING DOF FACTORs (CDFs), a family of rhythmically expressed transcriptional repressors known for their roles in the photoperiodic regulation of flowering time (Imaizumi et al., 2005). As LKP2 and its homolog FKF1 interact with GI to control the light-dependent degradation of CDF proteins, these observations provide independent evidence for a role of the LKP2/GI/CDF regulatory module in drought tolerance.

## Circadian Control of Hormone Biosynthesis and Signaling

Many plant responses to abiotic stress are mediated by phytohormones. For example, abscisic acid (ABA) accumulates rapidly upon exposure to water stress or salinity and controls many stress-adaptation responses (Agarwal and Jha, 2010). ABA levels are under circadian control. Peak levels were observed during the day in the angiosperms *Senecio minimus*, *Arbutus unedo*, and *Arabidopsis thaliana*, and in the gymnosperm *Callitris rhomboidea* (Burschka et al., 1983; Lee et al., 2006; McAdam et al., 2011). More complex temporal patterns have been observed in *Nicotiana tabacum*, where ABA levels showed a first peak immediately after dawn, a second in the afternoon and a third at night (Novakova et al., 2005). Circadian regulation takes place at the level of ABA biosynthesis. In *Arabidopsis*, the enzymes responsible for biosynthesis of carotenoid precursors and the rate-limiting enzyme *NINE-CIS-EPOXYCAROTENOID DIOXYGENASE* (NCED) peak in the morning (Thompson et al., 2000; Covington et al., 2008). In maize, the rhythmic expression of ABA biosynthetic enzymes was also consistent with ABA synthesis taking place in the early morning (Khan et al., 2010). In sugar cane, the temporal pattern of ABA biosynthesis may be more complex as one gene involved in ABA synthesis peaked in the early morning, and two others peaked during the night (Hotta et al., 2013). The rhythmic production of ABA was proposed to be controlled by the PRR5, 7, and 9 proteins, because analysis of a triple mutant (*prr5,7,9*) revealed increased ABA levels (Fukushima et al., 2009). However, this effect is likely to be indirect because ChIP-seq analyses of genome-wide binding sites for PRR5, PRR7, and TOC1 did not reveal binding of these proteins to the promoters of any component of ABA biosynthesis pathways (Table 1; Nakamichi et al., 2010; Huang et al., 2012; Liu et al., 2013).

**TABLE 1:** **Direct regulation of stress hormone biosynthesis and signaling by core components of the *Arabidopsis* circadian clock**.

**Hormone**	**Gene ID**	**Gene name**	**Role**	**TOC1 target**	**PRR5 target**	**PRR7 target**
JA	AT1G17420	LOX3	JA biosynthesis	Y	N	N
JA	AT1G72520	LOX4	JA biosynthesis	Y	N	N
JA	AT3G45140	LOX2	JA biosynthesis	Y	N	N
JA	AT1G19180	JAZ1	Repressor of JA-responsive genes	Y	N	N
JA	AT1G70700	JAZ9	Repressor of JA-responsive genes	Y	N	N
JA	AT5G11270	OCP3	JA signaling	Y	N	Y
JA	AT1G25540	PFT1	Regulator of JA responses	N	Y	N
JA	AT1G80840	WRKY40	JA-responsive transcription factor	N	Y	N
JA	AT3G06490	MYB108	JA-responsive transcription factor	N	Y	N
JA	AT1G52890	ANAC019	JA-responsive transcription factor	N	Y	N
ET	AT4G11280	ACS6	ET biosynthesis	Y	N	N
ET	AT5G03280	EIN2	ET signaling	Y	N	N
ET	AT5G47230	ERF5	ET-responsive transcription factor	Y	N	N
ET	AT1G53170	ERF8	ET-responsive transcription factor	Y	N	N
ET	AT4G39403	PLS	Negative regulator of ET signaling	N	Y	N
ABA	AT5G13630	ABAR	Putative ABA receptor	Y	Y	N
ABA	AT5G59220	PP2C:HAI1	Catalytic subunit of ABA receptor	N	Y	N
ABA	AT3G11410	PP2CA/AHG3	Catalytic subunit of ABA receptor	Y	Y	N
SA	AT2G41010	CAMBP25	Regulation of SA biosynthesis	Y	N	N
SA	AT1G20020	LFNR2	Regulation of SA biosynthesis	N	Y	N
SA	AT3G56710	SIB1	Regulation of SA biosynthesis	N	Y	N

Evidence for direct regulation of genes in the JA, SA, ET, or ABA pathways was obtained from genome-wide analyses of binding sites for the TOC1, PRR5, and PRR7 proteins (Huang et al., 2012; Nakamichi et al., 2012; Liu et al., 2013).

Plants’ ability to respond to ABA also varies with the time of the day. For example, stomatal closure is more sensitive to ABA in the afternoon, allowing for a more sensitive control of stomatal aperture (Correia et al., 1995). *TOC1*-overexpressing plants had widely open stomata throughout the diurnal cycle and exhibited increased sensitivity to drought. In contrast, plants with reduced expression of *TOC1* had the opposite phenotype, suggesting that *TOC1* may act to limit stomatal aperture (Legnaioli et al., 2009). This effect of *TOC1* was proposed to be mediated through the ABA-binding protein ABAR (also known as CHLH or GUN5) as expression of the *ABAR* gene was directly controlled by TOC1 binding to its promoter (Legnaioli et al., 2009; Huang et al., 2012). However, the function of ABAR in ABA signaling remains controversial as the mechanisms by which it regulates ABA responses are not known (Hubbard et al., 2010). TOC1 may nevertheless regulate ABA responses through a different signaling pathway, as ChIP-seq data indicate that it binds to the promoters of two class A phosphatases 2C, HAI1 and PP2CA/AHG3, that function as catalytic subunits of another ABA receptor [regulatory components of ABA receptor (RCAR), also known as pyrabactin resistance 1 (PYR) or PYR1-like (PYL); Huang et al., 2012].

The clock also regulates the expression of ABA-responsive genes in a direct manner. In *Arabidopsis*, 40% of ABA responsive genes were found to also be under circadian control (Covington et al., 2008). Analysis of motifs in the promoters of these genes revealed over-representation for LHY and CCA1 binding sites, suggesting that LHY and CCA1 control expression of large fraction of ABA-responsive genes by binding directly to their promoters (Huang et al., 2007).

While ABA is the main hormone involved in responses to drought and to osmotic stress, salicylic acid (SA), jasmonic acid (JA), ethylene (ET) also play a role in responses to other types of abiotic stress (Yang et al., 2004; Cheng et al., 2013; Wasternack and Hause, 2013; Kazan, 2015), and reduction of GA levels and signaling contributes to the resulting growth arrests (Colebrook et al., 2014).

Ethylene and JA act cooperatively in the regulation of responses to cold, salinity, drought and heat. The production of ET peaks during the subjective day in *Arabidopsis* and sorghum, and at the end of the day in potato (Finlayson et al., 1999; Thain et al., 2004; Chincinska et al., 2013). In *Arabidopsis*, rhythmic expression of the rate-limiting enzyme *ACC SYNTHASE 8 (ACS8)* is key to the rhythmicity of ET biosynthesis, and a knock-out mutation abolished circadian ET accumulation (Thain et al., 2004). Another *ACS* gene (*ACS6*) may also be under circadian control, as TOC1 was found to bind its promoter (Huang et al., 2012; Table 1). Two putative *ACC OXIDASE* enzymes may also play a role since they exhibit rhythmic expression with a similar phase as *ACS8* (Covington et al., 2008). Sucrose transport from source to sink tissue may contribute to the rhythmic production of ET, as mutation of the rhythmically expressed sucrose transporter *SUT4* abolished diurnal changes in ET levels in potatoes (Chincinska et al., 2013). The clock may also influence signaling downstream of ET, since the ethylene-responsive transcription factor (ERF) *ETHYLENE-INSENSITIVE 3* (*EIN3*) is expressed rhythmically in *Arabidopsis* under the control of the clock-associated *XAP5 CIRCADIAN TIMEKEEPER* (*XCT*; Ellison et al., 2011). Furthermore, ChIP-seq analyses revealed the binding of TOC1 and PRR5 proteins to genes encoding *ERFs* 8 and 5, and to regulators of ET signaling such as *EIN2* and *POLARIS* (*PLS*; Huang et al., 2012; Nakamichi et al., 2012; Table 1).

Jasmonic acid levels also peak in the middle of the day (Goodspeed et al., 2012; Zhang et al., 2013). Rhythmic changes in JA levels may be controlled by the TOC1 protein, as TOC1 was found at the promoters of several genes encoding 13-lipoxygenase enzymes (*13-LOX*) which initiate the biosynthesis of JA (Table 1). Responses to JA may also be regulated by TOC1, PRR5, and PRR7, as ChIP-seq analyses revealed binding of these proteins to the promoters of several genes encoding regulators of JA responses. This included two negative regulators of the JA signaling pathway (JASMONATE ZIM DOMAIN or JAZ proteins) and several JA-induced transcription factors (Huang et al., 2012; Nakamichi et al., 2012; Liu et al., 2013; Table 1). However, the gating of JA responses seems to be primarily mediated by the clock-associated protein TIME FOR COFFEE (TIC; Shin et al., 2012). TIC interacts with a positive regulator of JA signaling, MYC2, leading to its proteasomal degradation and to the down-regulation of JA responses (Kazan and Manners, 2013). *MYC2* mRNA accumulation is also under circadian control, providing another layer of rhythmic regulation of JA signaling. While *tic* mutants retain some level of gating of JA responses, this is completely abolished in *myc2* mutants (Shin et al., 2012).

Salicylic acid is increased in response to most biotic stresses and was proposed to have an acclimation-like effect, due primarily to enhanced anti-oxidative capacity (Horváth et al., 2007; Miura and Tada, 2014). SA is generally thought to act antagonistically with JA, at least with regard to defenses against pathogens (Spoel et al., 2007). As might be expected, SA levels peaks at night in *Arabidopsis*, 180°out of phase with JA levels (Goodspeed et al., 2012, 2013; Zhang et al., 2013). PRR5 and TOC1 may play a role to up-regulate the production of SA at night, as they bind to the promoters of several genes encoding regulators of SA biosynthesis (Huang et al., 2012; Nakamichi et al., 2012; Liu et al., 2013; Table 1). SA accumulation is negatively influenced by a rhythmically expressed phosphate transporter gene (*PHT4;1*) and this has also been proposed to contribute to oscillations in SA levels (Wang et al., 2014).

Altogether, these studies reveal a pervasive effect of the clock on stress-responsive hormonal pathways. Remarkably, circadian regulation occurs at multiple levels, including hormone biosynthesis, signaling and rhythmic expression of hormone-responsive genes. ChIP-seq analyses have revealed the direct regulation of key components of hormone signaling pathways by PRR5, PRR7, and TOC1 (Table 1) and it is likely that further genome-wide analyses will reveal additional roles for other components of the clock. Interestingly, data available so far reveal a division of labor between the PRR proteins. For example TOC1 was found at the promoter of several JA biosynthesis genes (LOX2,3,4) and of two repressors of JA responses (JAZ1,9) whereas PRR5 was found at a distinct set of genes encoding regulators of JA responses and JA-responsive transcription factors (PFT1, WRKY40, MYB108: ANAC019; see Table 1) and PRR7 only regulated one JA signaling component (OCP3). This may reflect a need for sequential expression of these genes, or alternatively, for independent control of their expression in response to changes in the environment. Understanding how regulation by the different clock proteins is integrated to regulate flux through the different signaling pathways will be a major challenge, which can only be addressed through mathematical modeling.

## Effects of Abiotic Stress on the Transcription of Clock Genes

While the clock has a clear impact on how plants respond to environmental stress, there is also evidence that the function of the clock is altered in response to abiotic stress conditions. For example, high salinity resulted in lengthening of the circadian period in wheat, and osmotic stress increased the expression level of barley clock genes and advanced their phase of expression (Erdei et al., 1998; Habte et al., 2014). Similarly, drought stress reduced the expression of evening-specific components of the clock in soybean (*TOC1*, *LUX*, *ELF4*, and *PRR*-like genes), and this led to disruption of the circadian system (Marcolino-Gomes et al., 2014). Although the period of the clock varies by less than 1 h in *Arabidopsis* over the physiological range of temperatures (Gould et al., 2006; Salome et al., 2010), the expression level of oscillator components is altered. Peak expression levels of the *TOC1* and *LUX* transcripts increase as temperatures get warmer, whereas *CCA1* and *LHY* RNA rhythms increase in amplitude and peak expression level as temperatures get colder, from 17 to 12°C (Gould et al., 2006; Mizuno et al., 2014). On the other hand, heat stress was reported to shorten the circadian period (Kusakina et al., 2014), whereas cold stress leads to a loss of rhythmicity. At 4°C, most clock-associated genes oscillate with reduced amplitudes under day-night cycles and become arrhythmic upon transfer to constant light. Only a small proportion of the genes that cycle at 20°C maintain clear diurnal oscillations at 4°C (Martino-Catt and Ort, 1992; Bieniawska et al., 2008; Espinoza et al., 2010).

These effects of abiotic stress on the function of the clock may be mediated by changes in the level of stress response hormones. In *Arabidopsis*, SA application did not influence circadian period length, but application of the ET precursor ACC (1-aminocyclopropane-1-carboxylicacid) shortened the period length (Hanano et al., 2006). In contrast, ABA treatment lengthened the period of the *Arabidopsis* clock (Hanano et al., 2006). This was associated with a reduction in *CCA1* mRNA levels, which is surprising because loss of function of *CCA1* causes a short period phenotype (Green and Tobin, 1999). However, ABA may affect the oscillator mechanism by altering expression of other clock genes, which would result in lengthening of period. Evolutionary conserved ABREs are present in the promoters of *TOC1*, *LHY*, and *CCA1*, and may represent multiple points of entry for ABA signals into the clock (Bieniawska et al., 2008; Spensley et al., 2009; Picot et al., 2010; Habte et al., 2014).

Several core clock components are regulated by stress-related transcription factors. For example, the heat shock transcription factor HsfB2b represses transcription from the *PRR7* promoter at high temperatures and in response to drought (Kolmos et al., 2014), and binding of CBF1 to the promoter of the *LUX* gene plays a role to maintain robust transcriptional oscillations at low temperatures (Chow and Kay, 2013). In addition, the transcriptional repressor FLOWERING BASIC HELIX-LOOP-HELIX 1 (FBH1) binds to the *CCA1* promoter. FBH1 expression is up-regulated by heat and down-regulated by cold, and its overexpression in transgenic plants leads to reduced expression of *CCA1* and shortening of circadian period by approximately 1 h. This suggest that, in wild-type plants, *FBH1* plays a role to down-regulate *CCA1* expression at warm temperatures (Nagel et al., 2014).

## Differential Splicing of Clock Genes in Response to Environmental Stress

The plant clock is also regulated by alternative splicing, and mutation of a spliceosomal component (*SPLICEOSOMAL TIMEKEEPER LOCUS1* or STIPL1) produced a long-period phenotype in *Arabidopsis* (Jones et al., 2012). Many clock-associated genes including *CCA1*, *LHY*, *PRR7*, *PRR9*, *TOC1*, *ELF3*, and *ZTL* undergo extensive alternative splicing through diverse mechanisms including intron retention, exon skipping and selection of alternative 5′ splice sites (Filichkin et al., 2010; Filichkin and Mockler, 2012; James et al., 2012; Seo et al., 2012; Kwon et al., 2014). Many of these alternative splicing events occur in response to abiotic stress conditions (Filichkin et al., 2010, 2015; Filichkin and Mockler, 2012; James et al., 2012; Seo et al., 2012; Kwon et al., 2014). For example, low temperatures suppress alternative splicing of *ELF3* but induce that of *TOC1*. In contrast, heat induces the alternative splicing of *CCA1*, *PRR7*, *TOC1*, and *ELF3* genes, and high salinity suppresses that of *ELF3* (Kwon et al., 2014).

Alternatively spliced isoforms often contain a premature termination codon that can give rise to a shorter but still functional protein, or to a non-functional protein that will be degraded by the non-sense-mediated decay pathway. For example, the *CCA1* and *LHY* transcripts are both alternatively spliced to form either the full-length protein or a truncated protein lacking the MYB DNA-binding region (James et al., 2012; Seo et al., 2012). Both truncated proteins, produced through intron retention, suppress the formation of functional homo- and heterodimers (CCA1-CCA1, CCA1-LHY, and LHY-LHY) through competitive inhibition. This impairs the ability of LHY and CCA1 proteins to bind DNA and results in dampened rhythmicity. Interestingly, the production of the truncated isoform of CCA1 (CCA1-β) decreases at low temperature, while that of truncated isoform of LHY increases. This results in a relatively constant level of functional homo- and heterodimers and enables the clock to maintain a constant period length across the physiological range of temperatures (James et al., 2012). Overexpression of the full-length form of CCA1 (*35S::CCA1-α*) enhanced freezing tolerance whereas overexpression of the truncated variant (*35S::CCA1*-β) decreased it (Seo et al., 2012). This was taken to suggest that the regulation of *CCA1* alternative splicing by cold temperatures is important for freezing tolerance. However, the effect of these transgenes is not an accurate reflection of what happens in response to temperature in wild-type plants, as any change in the splicing of CCA1 is normally compensated for by an opposite change in the splicing of LHY. An alternative and perhaps simpler interpretation for the effects of *CCA1-*α and *CCA1*-β overexpression is that reduced function of both LHY and CCA1 proteins in *35S::CCA1*-β plants may result in a cold-sensitive phenotype, whereas increased function of CCA1 in *35S::CCA1-*α may result in enhanced chilling resistance. More work is required to fully understand the importance of *CCA1* alternative splicing for plant tolerance to cold temperatures.

The molecular mechanisms linking the plant circadian clock to the control of RNA processing are largely unknown, but PROTEIN ARGININE METHYLTRANSFERASE 5 (PRMT5) is thought to play a key role in this process. This rhythmically expressed protein acts to transfer methyl groups to arginine residues present in histones and Sm spliceosomal proteins, and contributes to the regulation of many pre-messenger-RNA splicing events (Sanchez et al., 2010). In particular, *prmt5* mutant plants produce an alternatively spliced form of the core clock component *PRR9*, resulting in a long period phenotype (Hong et al., 2010; Sanchez et al., 2010; Hernando et al., 2015). The rhythmic expression of PRMT5 was responsive to both light and temperature cues, but interestingly the transcript accumulated at different phases in different environmental conditions. Expression of PRMT5 peaked at dusk under diurnal light–dark cycles, but peaked just before dawn under thermocycles (Hong et al., 2010). This altered phase in response to thermocycles was also seen when plants were exposed to photocycles suggesting that the PRMT5 rhythm is preferentially synchronized to temperature cycles. Thus, PRMT5 may play an important role to ensure changes in splicing of the clock genes in response to diurnal changes in temperature. Interestingly, *prmt5* mutants also exhibit reduced tolerance to salinity (Hernando et al., 2015), but it is not clear at this point whether this is related to the altered function of their circadian clock, or whether this is due to direct effects on abiotic stress response pathways.

Alternative splicing is known to mediate clock responses to environmental changes in other organisms. In *Neurospora*, temperature-dependent alternative splicing of the *FREQUENCY* transcript allows adaptation of the circadian clock to seasonal variations of photoperiod and temperature (Diernfellner et al., 2005, 2007). In *Drosophila*, thermosensitive splicing in the 3′-UTR of the clock gene *period* (*per*) leads to phase advanced accumulation of *per* RNA on cold days, which is crucial for seasonal adaptation of the circadian locomotor activity rhythm (Majercak et al., 1999). Interestingly, the *dart5-1* mutant, which is affected in the *Drosophila* PRMT5 homolog, also exhibited an abnormal period of locomotor activity (Sanchez et al., 2010). This suggested that PRMT5 may also play a role to regulate the alternative splicing of one or more clock genes in flies, and may identify a conserved mechanism for alternative splicing of clock-associated genes in response to environmental signals.

Abiotic stress may also affect the abundance of clock-associated proteins at the post-translational level. For example, salt stress induces the proteasomal degradation of the GI protein, thus reducing the amplitude of its oscillations (Kim et al., 2013). Reduced GI protein levels contribute to salt acclimation by freeing the SOS2 kinase to activate the Na^+^/H^+^ antiporter SOS1. It is likely that further examples of such post-translational regulation will emerge in the near future.

These findings are interesting because they show that the function of the circadian clock is altered in response to abiotic stress. Furthermore, the presence of evolutionarily conserved stress-responsive promoter elements in the promoters of clock genes from distantly related species suggests that these effects of abiotic stress on the clock mechanism may provide a selective advantage.

## Conclusions and Perspectives

**FIGURE 3:**
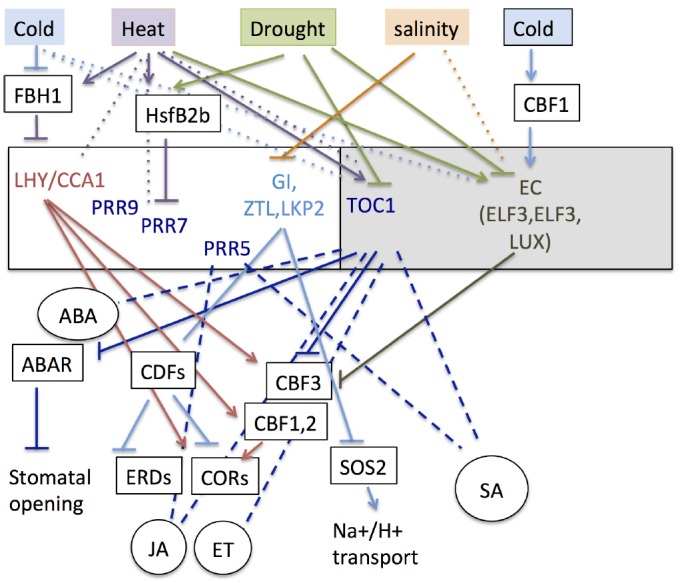
**Role of the circadian clock in integration of abiotic stress signals.** The white and shaded rectangles illustrate the day and night intervals of the circadian cycle. Oscillator components are listed within these boxes and their position in the rectangles gives an indication of the timing of their expression relative to each other and to dawn and dusk. The top part of the diagram illustrates the effects of different types of abiotic stress on expression of the clock genes. The bottom part of the diagram shows the effects of oscillator components on abiotic stress response pathways. Genes are indicated in boxes and hormones in ovals. Solid blunt and pointed arrows indicate transcriptional activation and repression, respectively. Dotted lines indicate induction of alternative splicing. Dashed lines indicate regulation of hormonal pathways. These lines have no sign because regulation of opposite sign may occur at different levels of the hormone biosynthesis and signaling pathway and the net effect of this regulation is not known.

The past few years of research have revealed a web of regulatory connections between abiotic stress response pathways and the plant circadian clock. The current knowledge of these interactions is summarized in Figure 3, but the picture is unlikely to be complete at this stage. Many aspects of abiotic stress responses are regulated directly by components of the central oscillator. The clock modulates the activity of stress response pathways at multiple levels and through a range of mechanisms, including the transcriptional regulation of key regulators of the stress response, the direct regulation of stress-responsive genes and signaling by stress response hormones. Most intriguingly, abiotic stress causes altered transcription and/or splicing of the clock genes, suggesting that abiotic stress response pathways feedback onto the oscillator to regulate its function.

How does circadian regulation of abiotic stress responses provide an advantage to plants, when their constitutive expression would provide a high level of stress tolerance at all times? As stress responses in plants are frequently associated with a growth arrest, we suggest that circadian regulation may provide an advantage by ensuring that plants can grow during part of the diurnal cycle, while only expressing stress tolerance mechanisms at times of the day when they are required most critically. However, this does not explain the contribution of the clock to salinity and osmotic stress tolerance, as these stress conditions do not vary in a diurnal fashion. Therefore, the clock may also contribute to abiotic stress tolerance via some other mechanisms.

As the clock allows plants to perceive changes in season (Carré, 2001; Yanovsky and Kay, 2002), it may act to modulate abiotic stress responses depending on the time of the year. This would ensure that a strong response is only triggered at those times of the year when a stress is likely to be pronounced and persistent. Thus, plant responses to cold were recently found to be regulated by seasonal changes in photoperiod (Lee and Thomashow, 2012). Under warm temperatures, the *CBF2* gene oscillated with higher amplitude under short day conditions (8L16D) than under long day conditions (16L8D). This resulted in up-regulation of the CBF pathway and increased freezing tolerance in the winter. Another study suggested that CDFs, a family of transcription factors known for their roles in the photoperiodic regulation of flowering, may mediate improved cold tolerance under short-day photoperiods. Short days lead to the stabilization of CDF proteins, which act to up-regulate expression of cold-responsive genes, resulting in enhanced cold tolerance in the winter (Fornara et al., 2015). Future work may reveal similar seasonal regulation of other types of abiotic stress responses in plants.

As the function of the clock is altered in response to abiotic stress, circadian regulation may also provide a form of memory of the signal, which may contribute to the stress acclimation process. Just like perturbing the oscillation of a pendulum results in lasting changes in its swinging pattern, acute up- or down-regulation of the clock genes in response to abiotic stress would be expected to result in persistent changes in the phase or amplitude of their oscillation. This may, in turn, result in long-term changes in the expression of the stress response pathways that they regulate. For example, water deficit causes an increase in the amplitude of the rhythmic expression of plasma membrane aquaporins, and this was proposed to benefit the plant by decreasing transpiration and by avoiding excessive dehydration of the rhizosphere that would dramatically decrease its hydraulic conductivity (Caldeira et al., 2014).

Figure 3 suggests a number of mechanisms by which the clock might ensure that stress responses are sustained following initial exposure. For example, cold temperatures increase the expression of *LHY* and *CCA1*, and this would in turn act to enhance expression of cold-responsive genes (*CBFs* and *CORs*) over multiple circadian cycles. Heat stress, on the other hand, down-regulates *LHY* and *CCA1* expression while increasing that of *TOC1*, and this would be expected to reduce the peak expression of *CBF* genes. Drought stress represses expression of *TOC1*, resulting in reduced stomatal opening, while salinity represses expression of *GI*, which promotes the activity of the Na^+^/H^+^ transporter SOS1. However, as plants in their natural environment are often exposed to multiple types of abiotic stresses simultaneously, the level and temporal pattern of expression of the clock genes is more likely to reflect the integration of multiple signals. For example, drought and heat stress, which are often experienced in combination, have opposite effects on *TOC1* and *LUX* transcription, but act synergistically to enhance *PRR7* expression by promoting that of HSFB2b. Therefore the predicted combined effect would be elevated expression of *PRR7* and intermediate levels of *TOC1* and *LUX* transcription. On the other hand, cold promotes the expression of *LUX* and CCA1 but has no known effect on *TOC1*. Therefore, the predicted combined effect of cold and drought would again be intermediate levels of *LUX* expression, but this would be associated with low levels of *TOC1* and high levels of *CCA1*. Thus, different combinations of environmental stresses may lead to distinct profiles of expression of the clock genes and to distinct effects on downstream stress responses. Further work will be required to test whether such integration of abiotic stress signals by the clock does take place, and whether it plays a role to maximize plant survival or productivity.

In summary, circadian regulation may provide a sophisticated, tunable mechanism to ensure an optimum compromise between plant growth and abiotic stress tolerance. Adjustments in clock function in response to changes in the environment may allow plants to re-adjust this balance and to ensure a sustained increase in abiotic stress tolerance when needed. If these hypotheses are correct, such mechanisms may in the future be exploited for improving abiotic stress tolerance in crops without reducing yield.

## Author Contributions

JG and CS carried out the initial research and wrote the first draft of the manuscript. JG prepared Figure 1, and CS Figure 2 and Table 1. IC edited the work, wrote the conclusions and perspectives section, prepared Figure 3 and finalized the manuscript.

### Conflict of Interest Statement

The authors declare that the research was conducted in the absence of any commercial or financial relationships that could be construed as a potential conflict of interest.
